# Complexity of nursing care at 24 h from admission predicts in-hospital mortality in medical units: a cohort study

**DOI:** 10.1186/s12913-020-5038-5

**Published:** 2020-03-06

**Authors:** Davide Ausili, Davide Paolo Bernasconi, Paola Rebora, Lucia Prestini, Giorgio Beretta, Laura Ferraioli, Anna Cazzaniga, Maria Grazia Valsecchi, Stefania Di Mauro

**Affiliations:** 1 School of Medicine and Surgery, University of Milano-Bicocca, Monza, Italy; 2 Center of Biostatistics for Clinical Epidemiology, School of Medicine and Surgery, University of Milano-Bicocca, Monza, Italy; 3 ASST della Valtellina e dell’Alto Lario, Sondrio, Italy; 4 ASST di Lecco, Lecco, Italy

**Keywords:** Hospitals, Hospital medicine, Hospital information systems, Health services research, Quality of health care, Nurse staffing, Nursing care, Patient-centered care

## Abstract

**Background:**

The Informative System of Nursing Performance was developed to measure complexity of nursing care based on the actual interventions performed by nurses at the point of care. The association of this score with in-hospital mortality was not investigated before. Having this information is relevant to define evidence-based criteria that hospital administrators can use to allocate nursing workforce according to the real and current patients’ need for nursing care. The aim of this study is to assess the association between complexity of nursing care and in-hospital mortality.

**Methods:**

Register-based cohort study on all patients admitted to acute medical wards of a middle-large hospital in the North of Italy between January 1, 2014, to December 31, 2015 and followed up to discharge. Out of all the eligible 7247 records identified in the Hospital Discharge Register, 6872 records from 5129 patients have been included. A multivariable frailty Cox model was adopted to estimate the association between the Informative System of Nursing Performance score, both as continuous variable and dichotomized as low (score < 50) or high (score ≥ 50), and in-hospital mortality adjusting for several factors recorded at admission (age, gender, type of admission unit, type of access and Charlson Comorbidity Index).

**Results:**

The median age of the 5129 included patients was 76 [first-third quartiles 64–84] and 2657(52%) patients were males. Over the 6872 admissions, there were 395 in-hospital deaths among 2922 patients at high complexity of nursing care (13.5%) and 74/3950 (1.9%) among those at low complexity leading to a difference of 11.6% (95% CI: 10.3–13.0%). Adjusting by relevant confounders, the hazard rate of mortality in the first 10 days from admission resulted 6 times significantly higher in patients at high complexity of nursing care with respect to patients at low complexity (hazard ratio, HR 6.58, 95%CI: 4.50;9.62, *p* < 0.001). The HR was lower after 10 days from admission but still significantly higher than 1. By considering the continuous score, the association was confirmed.

**Conclusion:**

Complexity of nursing care is strongly associated to in-hospital mortality of acute patients admitted to medical departments. It predicts in-hospital mortality better than widely used indicators, such as comorbidity.

## Background

In-hospital mortality is an important indicator of the quality and safety of hospital care worldwide [1, 2]. Understanding predictors of in-hospital mortality is relevant to assess and to improve the quality and safety of provided care [3]. Several studies explored those predictors focusing on patients’ clinical characteristics. Comorbidity [4], clinical instability [5] and baseline functional status [6] were found to be strong predictors of in-hospital mortality. Other studies focused on the severity of a specific admission disease or condition. For example, the New York Heart Association class [7], the QRS voltage [8] and the presence of at least one comorbidity [9] were found to be predictors of in-hospital mortality in patients with acute heart failure or acute myocardial infarction. An increasing number of studies recently focused on hospital organization variables. The accuracy of medical transport information [10] and the time to disposition plan in emergency department [11] are examples of organizational factors that affect in-hospital mortality. Overall, the majority of the studies that focused on hospital organization investigated nursing-related variables.

Nurse staffing levels [12], nursing skill mix [13], nurse education [14], and the quality of work environment [15], were found to be predictors of in-hospital mortality. All together, these results suggest that nursing care has a strong impact on both patient safety and quality of hospital care [16, 17]. Thus, it is extremely relevant that hospital administrators provide adequate nursing workforce and work environments [18]. However, recent cross-national investigations showed that only few hospitals are able to maintain recommended nurse staffing levels [19], that nursing workforce is aging [20] and that the lack of resources [21, 22] and the shortage of nurses are common issues for many countries [23]. In this worrying context, being able to identify those patients that are more complex from the nursing point of view and that require higher levels of nursing care, could be valuable for hospital administrators in order to allocate resources, to distribute the nursing workforce on the base of patients’ complexity, and to improve hospital safety and quality of care.

In Italy, Diagnosis Related Groups (DRGs) are in use for nurse staffing purposes, assuming that medical diagnosis determines the amount of nursing care needed [24]. The Informative System of Nursing Performance score (SIPI – *Sistema Informativo della Performance Infermieristica*) was developed in 2012 by a group of Italian researchers with the aim of measuring the complexity of nursing care [24]. This classification system was built with the aim to assess the demand for nursing care directly on patients’ needs, rather than indirectly on their medical diagnosis.

Overall, the SIPI validation study showed promising results [24]. However, the association between the complexity of nursing care and in-hospital mortality– as one of the main quality of care indicators - remains unexplored while it could have crucial implications on the organization of hospital care and the development of effective strategies to allocate nursing workforce and to improve patient safety.

The aim of this study was to assess the association between the complexity of nursing care, as measured by the SIPI score at 24 h from admission, and in-hospital mortality of patients admitted to an acute medical department.

## Methods

A register-based retrospective cohort study [25] has been conducted in a medium-large hospital in the North of Italy where the SIPI score has been included in 2013 within the standard clinical patient electronic documentation with the aim of describing the complexity of nursing care within usual administrative information. Authorization was obtained by the Institutional Review Board of the participating hospital. An informed consent was signed by each patient at the admission to the hospital for the use of sociodemographic, clinical, and administrative data for epidemiological purposes.

### Sample

All in-hospital patients dismissed from the acute medical department in a medium-large hospital in the North of Italy from January 1st 2014 to December 31st 2015 were included in the study. The only exclusion criterion was the inappropriate admission to the medical department due to the lack of available beds in the surgical department (i.e. patients that underwent or that were awaiting for surgery, extraordinarily and temporary admitted in the medical department while awaiting for their bed in a surgical ward). The medical department included 8 medical generalist and specialist clinical units. According to the local law for the accreditation of the hospitals, one of these units had a nurse to patient ratio of 1:10 and a nurse to support worker ratio of 1:1. The other seven units had a nurse to patient ration of 1:12 and a nurse to support worker ratio of 1:1 in the day shifts, and 1:0 in the night shift. In this study, we referred to the first one as “Medium Intensity” ward, and to the others as “Low Intensity” wards.

### Outcomes and measures

Sociodemographic and clinical characteristics of the sample were collected by the Hospital Discharge Register, together with survival status at discharge. These variables included: age, gender, type of unit where the patient was admitted (low or high intensity), type of admission (urgent or planned), length of stay and comorbidity. Comorbid conditions were measured by the use of the Charlson Comorbidity Index (CCI) [26]. The CCI is a widely used tool that assess the presence of 19 common diseases. Higher CCI scores indicate higher comorbidity. Its validity in the Italian population is supported by previous results [27, 28].

Complexity of nursing care was measured by the SIPI [24]. The SIPI consists of 18 binary items, indicating the presence/absence of a condition. These items refer to the following area of nursing activities: to assure breathing (2 items), to assure feeding and hydration (2 items), to assure urination and defecation (2 items), to assure hygiene (2 items), to assure mobility (3 items), to monitor cardiac function (1 item), to conduct diagnostic procedures (2 items), and to apply therapeutic procedures (4 items). The questionnaire has to be completed based on the nursing file of performed activities in the last 24 h. The SIPI score ranges from 0 to 100, with higher values indicating higher complexity of nursing care. The SIPI score was validated using a large multicentre cross-sectional design that involved 25 hospitals and more than 17,000 patients in the North of Italy. In this validation study, nurses that administered the SIPI were asked to indicate their own perception of the complexity of nursing care for each patient using a four classes scale (very low, medium-low, medium-high, very high). These classes, completed by nurses before administering the SIPI, were used as gold standard to evaluate the performance of the SIPI score [24]. A cut-off value of 50 points was chosen with high sensitivity and specificity (respectively 85 and 80%) to identify patients at high level of complexity [24]. Full information about the nursing activities measured by the SIPI can be retrieved in its validation paper [24].

In this study, the SIPI score was retrieved from the SIPI database where the complexity of nursing care is documented for each admitted patient at regular time intervals, by nurses working in the medical department. The first measurement at 24 h from the hospital admission has been used in this study to assess prospectively the association between the SIPI score at admission and in-hospital mortality.

### Statistical analysis

Patients’ characteristics at the first admission included in the final database were described using number and percentages for categorical variables and median and interquartile range (IQR) for quantitative variables. Comparisons between high and low complexity groups were made using the Fisher test and the Mann-Whitney test, as appropriate. We estimated the hazard of mortality since time of admission using a smoothed non-parametric estimator [29] both on the overall sample and stratified by SIPI > 50 or ≤ 50. The average mortality rate over the whole length of stay was also computed and compared between the SIPI groups. To estimate the association between the SIPI score and in-hospital mortality we fit uni- and multivariable Cox models adjusting for several factors recorded at admission [age, gender, type of access (urgent vs ordinary), type of ward (low intensity Vs medium intensity) and Charlson Comorbidity Index]. To account for the presence of multiple admissions for each patient we included a gamma frailty term in the model. Moreover, since the proportional hazard assumption was not tenable for SIPI, we estimated the corresponding hazard ratio within two time intervals: before and after day 10 since admission. The same regression analysis was also done considering the continuous SIPI score. As a sensitivity analysis, analogous Cox regression models were fitted only on data of patients with one single admission included in the final database. Significance level was set at 0.05, tests were two-sided and analyses were performed with R software version 3.5.1 with the following packages: “survival”, “prodlim”, “survivalROC” and “bshazard”.

## Results

**Fig. 1:**
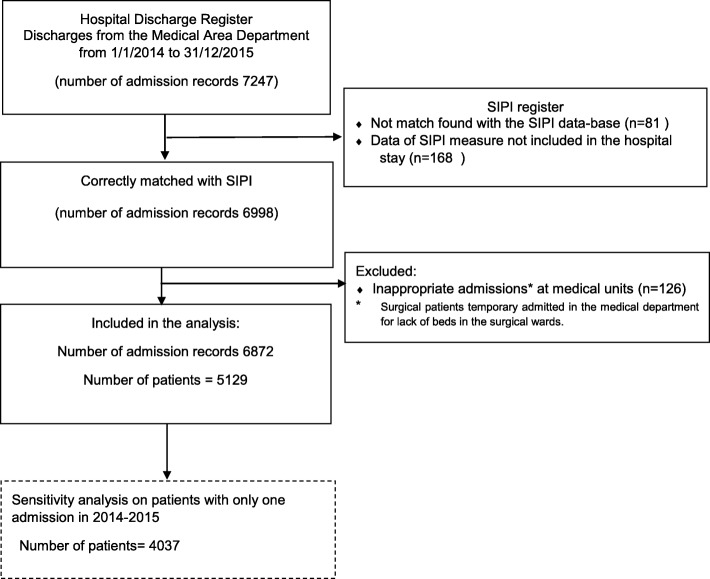
Flow-chart of the hospital admission records and the number of patients analysed in the study. The dashed box represents the data set used for sensitivity analysis

From the 7247 records identified in the Hospital Discharge Register, 6988 had a SIPI measure within 24 h from hospital admission. Among these, we excluded 126 patients that were inappropriately admitted to medical units (i.e. surgical patients awaiting for surgery), getting a total of 6872 records. These records referred to 5129 patients, as 1092 patients have been hospitalised multiple times in the period of interest (Fig. 1). The total of 5129 patients (median age 76 years IQR [64–84], 2657 (52%) males) that were admitted at least once to the acute medical department from January 1st 2014 to December 31st 2015 were included in this study. In the period of interest, the 5129 patients experienced 6872 hospital admissions with 724 patients (14.1%) having 2 admissions, 368 (7.2%) having 3 to 9 admissions and the remaining (4037, 78.7%) one admission only. The admission characteristics by SIPI value (≤50 versus > 50) are reported in Table 1. Patient’s admissions classified as “high nursing complexity” by the SIPI score (*n* = 2922, 42.5%) were older, had more comorbidities and had a higher percentage of females and urgent hospital access. Moreover, the corresponding hospital stay was significantly longer. Similar results were obtained when 5129 patients are analysed at their first hospital admission (Supplementary Table 1).

**Table 1:** Characteristics of the patients at the 6872 admissions and the ward and duration of hospital stay and status at discharge

**Variables**	**SIPI ≤ 50** *N* = 3950 (57.5%)	**SIPI > 50** *N* = 2922 (42.5%)	***P*value**
**At admission**			
Age, years (median [IQR])	70.50 [57.00, 79.00]	81.00 [73.00, 87.00]	< 0.001
Sex, Male, No.(%)	2233 (56.5)	1414 (48.4)	< 0.001
Charlson Comorbidity Index, No.(%)			
0	1109 (28.1)	568 (19.4)	< 0.001
1	2054 (52.0)	1354 (46.3)	
2	625 (15.8)	730 (25.0)	
3	141 (3.6)	239 (8.2)	
4	21 (0.5)	29 (1.0)	
5	0	2 (0.1)	
Ward, Low intensity, No.(%)	2577 (65.2)	1953 (66.8)	0.175
Access, Urgency, No.(%)	3454 (87.4)	2845 (97.4)	< 0.001
**At discharge**			
Length of stay, days (median [IQR])	9.00 [6.00, 15.00]	13.00 [8.00, 21.00]	< 0.001
Number of deaths, No.(%)	74 (1.9)	395 (13.5)	< 0.001

*IQR* interquartile range, *SIPI* Informative System of Nursing Performance

**Fig. 2:**
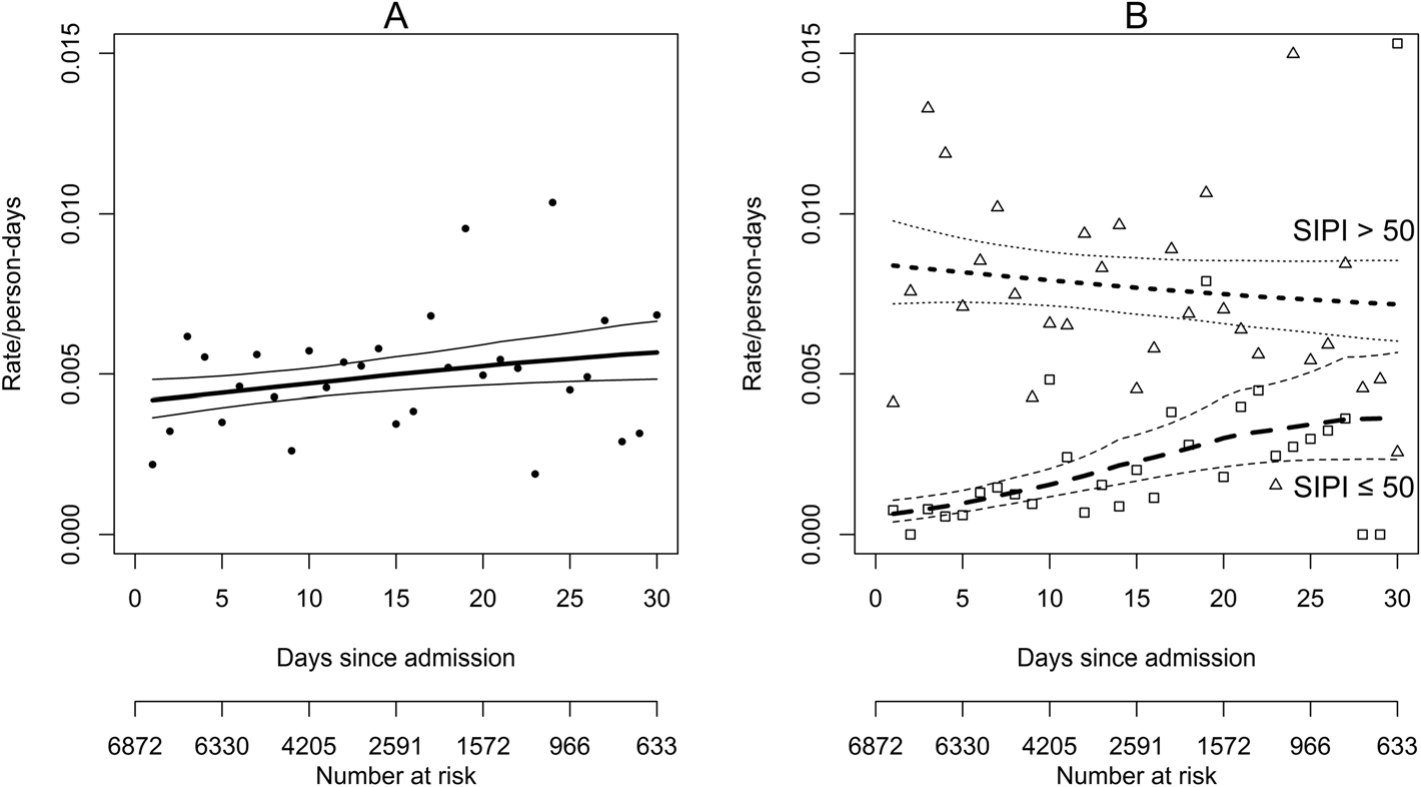
Estimated hazard rates: **a**) overall, **b**) stratified by SIPI≤50 or > 50. In both panels, dots represent the raw hazard estimates and lines represents a non-parametric smoothing function (thin lines are 95% confidence limits). In panel **b**, squares and dashed lines refer to admissions with SIPI≤50 while triangles and dotted lines refer to admissions with SIPI> 50

Over the 6872 admissions, there were 469 in-hospital deaths (6.8%), 395/2922 (13.5%) among those with a SIPI score ≥ 50 and 74/3950 (1.9%) among those with a SIPI score < 50 leading to a difference of 11.6% (95% confidence interval (CI): 10.3–13.0%). The in-hospital hazard rate by time since admission is reported in Fig. 2 panel A. The overall average mortality rate was 4.8 over 1000 person-days (i.e. about 5 deaths every 100 patients who stayed for 10 days). Among patients with a SIPI score at admission higher/equal and lower than 50 the average mortality was 7.8 and 1.6 over 1000 person-days, respectively. The SIPI score at admission showed a significant association with in-hospital mortality both in univariable and multivariable analysis (*P* < 0.0001) where adjustment for other risk factors was considered in a Cox regression model. The estimated hazard rate of mortality in the first 10 days from admission resulted 6 times higher in patients with SIPI> 50 compared to patients with SIPI≤50 (hazard ratio (HR) = 6.58, 95%CI: 4.50;9.62, *p* < 0.001), as shown in Table 2. The ratio between the two hazards was lower after 10 days from admission but still significantly higher than 1 (HR = 2.58, 95%CI: 1.83;3.64, *p* < 0.001). By considering the continuous SIPI score in the multivariable Cox model, the hazard rate of mortality in the first 10 days from admission resulted 1.6 times higher every 10 units of the SIPI score (HR = 1.633, 95% CI: 1.519;1.756, *P* < 0.001), and nearly 1.3 times higher after the 10 days from admission (HR = 1.255,95%CI: 1.176;1.339, *P* < 0.001). Results on patients with only one admission (sensitivity analysis, *n* = 4037) were consistent with the main analysis (Supplementary Table 2).

**Table 2:** Unadjusted and adjusted Cox models with gamma frailty (total number of events: 469) on mortality in 6872 hospital admissions. The HR of SIPI (> 50 vs ≤50) was estimated separately within 2 time-intervals: before and after day 10 since admission. The effect of SIPI as a continuous variable estimated on a separate multivariable model is also reported

**Factors**	**Unadjusted** (*N* = 6872)		**Adjusted** (*N* = 6872)	
	**HR (95% CI)**	***P*value**	**HR (95% CI)**	***P*value**
Age, per 10 years	1.483 (1.363;1.613)	< 0.001	1.233 (1.127;1.350)	< 0.001
Gender, Male vs Female	1.050 (0.875;1.260)	0.640	1.266 (1.051;1.527)	0.013
Ward, Medium Intensity vs Low	1.612 (1.333;1.949)	< 0.001	1.638 (1.348;1.991)	< 0.001
Access, Emergency vs Ordinary	5.037 (2.086;12.160)	< 0.001	2.732 (1.125;6.637)	0.026
CCI index, 1 vs 0	1.609 (1.238;2.091)	< 0.001	1.606 (1.235;2.089)	< 0.001
CCI index, ≥2 vs 0	1.654 (1.252;2.186)	< 0.001	1.258 (0.950;1.665)	0.110
SIPI > 50, vs ≤50 at ≤10 days since admission	7.907 (5.450;11.473)	< 0.001	6.576 (4.496;9.617)	< 0.001
SIPI > 50, vs ≤50 at > 10 days since admission	2.868 (2.048;4.016)	< 0.001	2.583 (1.831;3.644)	< 0.001
SIPI, per 10 points in the continuous SIPI score at ≤10 days since admission	1.634 (1.526;1.749)	< 0.001	1.633 (1.519;1.756) ^a^	< 0.001^a^
SIPI, per 10 points in the continuous SIPI score at > 10 days since admission	1.260 (1.183;1.341)	< 0.001	1.255 (1.176;1.339) ^a^	< 0.001^a^

^a^estimated on a separate multivariable model with the same covariates (regression coefficients not shown)

*HR* hazard ratio, *CI* confidence interval, *CCI* Charlson Comorbidity Index, *SIPI* Informative System of Nursing Performance

## Discussion

To the best of our knowledge, this is the first study assessing the association between complexity of nursing care at 24 h from the admission and in-hospital mortality of acute medical patients. We found that complexity of nursing care is strongly associated to in-hospital mortality of acute patients admitted to medical departments, even when the association is adjusted for other well known potential risk factors. Complexity of nursing care predicted in-hospital mortality surprisingly better than the widely used indicator of comorbidity (as measured by the Charlson Comorbidity Index) [26]. This might be due to the fact that the SIPI score is a more specific index of patients’ care needs, as measured by the activities that nurses perform in a day.

We found that 469 patients (6.8%) died during their hospital stay, out of the 6872 observed admissions in the medical department. This result is almost identical to previous longitudinal findings reporting in-hospital mortality among 12 Italian medical units [30], and suggests that performances of our centre are comparable to others, supporting also the generalizability of our results. Patients at high complexity of nursing care at their admission have a clinically significant increase in the hazard of dying during their hospital stay when compared with those at low complexity of nursing care. This association is particularly evident when considering the early period (10 days) after admission, where the adjusted mortality rate among patients deserving complex nursing care is at least 4 times higher (lower limit of the 95% confidence interval, with and estimated hazard ratio of more than 6) than that of less demanding patients. This result is extremely relevant for the administration of acute hospital medical departments. In fact, although the association between nurse staffing and mortality was previously demonstrated [12], a criterion to allocate nursing resources based on patient’s characteristics represents a relevant gap in this field. Of course, maintaining an adequate nurse to patient ratio is recommended for medical and surgical hospital settings, based on the results of Aiken and colleagues [12–17]. However, when this is not possible - as documented by previous cross-national surveys on nurse staffing [13, 19] in Europe and United States - the SIPI score can be used in medical departments to identify those patients that are more complex, that are at risk of dying, and that, consequently, require an increased amount of nursing care. In fact, as patients with SIPI > 50 have an increased hazard of dying, nursing surveillance is relevant to prevent failures to rescue, when severe complications can be prevented, or to assure a dignified dying, when those complications cannot be avoided [31]. Finally, the prevalence of patients with high nursing complexity (40%), was in line with the previous multi-centre Italian study in which it resulted nearly 50% [24]. This finding confirms that almost half of the patients is complex and demands relevant amount of nursing care.

The retrospective, mono-country and mono-centric nature of the study represents the main limitation of this research. Furthermore, data about nursing-related variables that were shown to be associated with patients’ mortality (i.e. nurse staffing, skill mix, and education), were not collected in this study. Thus, we were not able to adjust for these variables while assessing the association between the SIPI score at 24 h from admission and in-hospital mortality. However, the large sample size (both records and patients), the inclusion of all consecutive admissions, the use of validated administrative data to measure patients’ characteristics and mortality, the agreement between the principal and the sensitivity statistical analyses, support the validity of our results.

## Conclusion

We demonstrated that complexity of nursing care as measured by the SIPI at 24 h from the admission is a strong predictor of in-hospital mortality in acute patients admitted to medical departments. Hospital administrators can implement the SIPI measure to identify those patients, units or departments that require a major amount of nursing care, particularly when recommended staffing levels cannot be assured due to lack of resources. Future studies should: assess the association between complexity of nursing care and in-hospital mortality in surgical settings; assess the association between the complexity of nursing care measured by the SIPI score and in-hospital mortality, adjusting for nursing-related variables as staffing, skill mix and education; assess the relationship between complexity of nursing care and widely used early warning scores such as the National Early Warning Score [5]; identify the different levels of nurse staffing according to the complexity of nursing care as measured by the SIPI; assess the association between Diagnosis Related Groups (DRGs) and complexity of nursing care, in order to investigate if the current approach in allocating resources takes into account the demand of nursing care.

## Supplementary information

**Additional file 1: Table S1.** Characteristics of the 5129 patients at the first admission. **Table S2.** Unadjusted and adjusted Cox models (total number of events: 312) on mortality in patients with only one admission. The HR of SIPI (> 50 vs ≤50) was estimated separately within 2 time-intervals: before and after day 10. The effect of SIPI as a continuous variable estimated on a separate multivariable model is also reported.

## Data Availability

De-identified participant data that support the findings of this study may be obtained from the corresponding author upon reasonable request and after authorization of the local health authority.

## Abbreviations

CCI: Charlson Comorbidity Index
CI: Confidence interval
DRGs: Diagnosis Related Groups
HR: Hazard ratio
IQR: Interquartile range
SIPI: Informative System of Nursing Performance
