# Supplementary material for: Complexity of nursing care at 24 h from admission predicts in-hospital mortality in medical units: a cohort study
Source: BMC Health Serv Res. 2020 Mar 6;20:181. doi: 10.1186/s12913-020-5038-5 (PMC7059664; doi:10.1186/s12913-020-5038-5)
Supplement: Supplementary file 1 — Additional file 1: Table S1. Characteristics of the 5129 patients at the first admission. Table S2. Unadjusted and adjusted Cox models (total number of events: 312) on mortality in patients with only one admission. The HR of SIPI (> 50 vs ≤50) was estimated separately within 2 time-intervals: before and after day 10. The effect of SIPI as a continuous variable estimated on a separate multivariable model is also reported. [file 12913_2020_5038_MOESM1_ESM.docx]

**Supplementary Table 1.** Characteristics of the 5129 patients at the first admission.

| **Variables** | **SIPI ≤ 50**  N=3060 (59.7%) | **SIPI > 50**  N=2069 (40.3%) | **P value** |
| --- | --- | --- | --- |
| **At admission** |  |  |  |
| Age, years (median [IQR]) | 71.00 [55.75, 80.00] | 82.00 [75.00, 88.00] | <0.001 |
| Sex, Male, No.(%) | 1729 (56.5) | 928 (44.9) | <0.001 |
| Charlson Comorbidity Index , No.(%)  0  1  2  3  4  5 | 984 (32.2)  1511 (49.4)  455 (14.9)  98 (3.2)  12 (0.4)  0 | 466 (22.5)  936 (45.2)  491 (23.7)  160 (7.7)  15 (0.7)  1 (0.05) | <0.001 |
| Ward, Low intensity, No.(%) | 1928 (63.0) | 1342 (64.9) | 0.185 |
| Access, Urgency, No.(%) | 2771 (90.6) | 2031 (98.2) | <0.001 |
| **At discharge** |  |  |  |
| Length of stay, days (median [IQR]) | 9.00 [6.00, 15.00] | 13.00 [8.00, 21.00] | <0.001 |
| Number of deaths, No.(%) | 45 (1.5) | 267 (12.9) | <0.001 |

IQR: interquartile range; SIPI: Informative System of Nursing Performance

**Supplementary Table 2**. Unadjusted and adjusted Cox models (total number of events: 312) on mortality in patients with only one admission. The HR of SIPI (>50 vs ≤50) was estimated separately within 2 time-intervals: before and after day 10. The effect of SIPI as a continuous variable estimated on a separate multivariable model is also reported.

| **Factors** | **Unadjusted (N=4037)** | | **Adjusted (N=4037)** | |
| --- | --- | --- | --- | --- |
|  | **HR (95% CI)** | **P value** | **HR (95% CI)** | **P value** |
| Age, per 10 years | 1.591 (1.431;1.769) | <0.001 | 1.233 (1.127;1.350) | <0.001 |
| Gender, Male vs Female | 0.920 (0.737;1.149) | 0.464 | 1.266 (1.051;1.527) | 0.013 |
| Ward, Medium Intensity vs Low | 1.606 (1.276;2.022) | <0.001 | 1.638 (1.348;1.991) | <0.001 |
| Access, Emergency vs Ordinary | 5.847 (1.455;23.490) | 0.013 | 2.732 (1.125;6.637) | 0.026 |
| CCI index, 1 vs 0 | 1.666 (1.226;2.263) | 0.001 | 1.606 (1.235;2.089) | <0.002 |
| CCI index, ≥2 vs 0 | 2.034 (1.468;2.818) | <0.001 | 1.258 (0.950;1.665) | 0.110 |
| SIPI >50, vs ≤50 at ≤10 days since admission | 10.836 (6.550;17.929) | <0.001 | 6.576 (4.496;9.617) | <0.001 |
| SIPI >50, vs ≤50 at >10 days since admission | 3.025 (1.182;7.741) | 0.021 | 2.583 (1.831;3.644) | <0.001 |
|  |  |  |  |  |
| SIPI, per 10 points in the continuous SIPI score at ≤10 days since admission | 1.634 (1.526;1.749) | <0.001 | 1.633 (1.519;1.756)^*^ | <0.001 ^*^ |
| SIPI, per 10 points in the continuous SIPI score at >10 days since admission | 1.260 (1.183;1.341) | <0.001 | 1.255 (1.176;1.339) ^*^ | <0.001 ^*^ |

^*^ estimated on a separate multivariable model with the same covariates (regression coefficients not shown)

HR: hazard ratio; CCI: Charlson Comorbidity Index; SIPI: Informative System of Nursing Performance; CI: confidence interval.
